# Draft Genome Sequence of *Listeria monocytogenes* Strain LI0521 (syn. HPB7171), Isolated in 1983 during an Outbreak in Massachusetts Caused by Contaminated Cheese

**DOI:** 10.1128/genomeA.00729-14

**Published:** 2014-07-24

**Authors:** Arthur W. Pightling, Min Lin, Franco Pagotto

**Affiliations:** a Listeriosis Reference Service, Microbiology Research Division, Bureau of Microbial Hazards, Food Directorate, Health Products and Food Branch, Health Canada, Ottawa, Ontario, Canada; b Ottawa Laboratory Fallowfield, Canadian Food Inspection Agency, Ottawa, Ontario, Canada

## Abstract

*Listeria monocytogenes*, a pathogenic food-borne bacterium, is the causative agent of both sporadic and outbreak cases of human listeriosis. Here, we present the genome sequence of *L. monocytogenes* reference strain LI0521, isolated during an outbreak involving contaminated cheese, which has been used as the model during several proteomic studies.

## GENOME ANNOUNCEMENT

*Listeria monocytogenes*, a Gram-positive pathogenic food-borne bacterium, is the causative agent of sporadic and outbreak cases of human listeriosis (1, 2). Although naturally occurring in plant, soil, and surface water environments, when present in the food supply and ingested by humans through contaminated, ready-to-eat foods, *L. monocytogenes* can cause severe and life-threatening illness.

Listeriosis may result in central nervous system infections, bacteremia and endocarditis, especially among immunocompromised or elderly adults, while listeriosis that occurs during pregnancies may result in abortions or stillbirths (3). Although its genome sequence was undetermined, *L. monocytogenes* serovar 4b strain LI0521 (syn. HPB7171) has been the subject of several studies since its isolation in 1983 during an outbreak caused by contaminated cheese in Massachusetts (4). These studies include the identification of diagnostic targets for isolation and detection of viable *L. monocytogenes* from test samples (5) and the characterization of N-acetylglucosaminidae activity and its role in the virulence of a surface autolysin, IspC (6, 7, 8), the humoral immune response against *Listeria* infections (9), and the development of monoclonal antibodies to surface antigens (10).

To make the genome sequence of the LI0521 strain available for further experimental studies, short-read sequence data were generated by indexing with the Nextera XT DNA sample preparation kit (Illumina, San Diego, CA) and sequencing the genome on a MiSeq Benchtop sequencer (Illumina) for 500 cycles. Quality trimming and filtering of the reads were performed with the trim-fastq.pl module in the Popoolation v1.2.2 (11) package to a minimum length of 75 bp and *Q* score of 30, yielding a total of 3,861,102 reads. Reads were then assembled *de novo* into a high-quality draft genome with VelvetOptimiser v2.2.5 (http://vicbioinformatics.com) running Velvet v1.2.10 (12), resulting in 34 non-overlapping contigs with a total length of 3,005,759 bp, a 37.8% G+C content, and 231-fold sequencing coverage. Gene predictions and annotations were performed with Prokka: Prokaryotic Genome Annotation System v1.7 (http://vicbioinformatics.com); a total of 3,133 features were identified, including 2,971 open reading frames, 44 tRNA genes, 1 transfer-messenger RNA gene, approximately 100 miscellaneous non-coding RNAs, the temperate phage PSA (13), and 38 pseudogenes. *In silico* analysis of the multi-locus sequence typing (14) targets (*abcZ, bglA, cat, dapE, dat, ldh*, and *lhkA*) indicate that this strain is closely related to lineage I clonal complex 2 (CC2), sequence type 2 (ST2). In addition, the LI0521 genome includes at least 37 putative internalin coding sequences, including *inlA, inlB, inlJ*, and a conserved *prfA* virulence gene cluster (15). The AscI and ApaI pulsed-field gel electrophoresis (PFGE) patterns are 0038 and 0031, respectively, and the multi-locus variable number tandem repeat analysis (MLVA) allele string is 00-02-16-24-16-12-05-14 (4).

### Nucleotide sequence accession numbers.

This whole-genome shotgun project has been deposited at DDBJ/EMBL/GenBank under the accession no. JMMW00000000. The version described in this paper is the first version, JMMW01000000.
